# Evaluation of electrocardiographic P wave parameters in predicting long-term atrial fibrillation in patients with acute ischemic stroke

**DOI:** 10.1055/s-0042-1755322

**Published:** 2022-11-09

**Authors:** Tufan Çinar, Mert İlker Hayiroğlu, Murat Selçuk, Göksel Cinier, Vedat Çiçek, Selami Doğan, Şahhan Kiliç, Süha Asal, Murat Mert Atmaca, Ahmet Lütfullah Orhan

**Affiliations:** 1 Health Sciences University, Sultan II, Abdülhamid Han Training and Research Hospital, Department of Cardiology, Istanbul, Turkey.; 2 Health Sciences University, Dr. Siyami Ersek Training and Research Hospital, Department of Cardiology, Istanbul, Turkey.; 3 Health Sciences University, Sultan II, Abdülhamid Han Training and Research Hospital, Department of Neurology, Istanbul, Turkey.

**Keywords:** p Wave, Reference Parameters, Electrocardiography, Ischemic Stroke, Onda p, Parâmetros de Referência Eletrocardiografia, AVC Isquêmico

## Abstract

**Background** Electrocardiographic parameters, such as P wave peak time (PWPT), P wave duration (PWD), and P wave amplitude in lead DI, have been utilized to assess left atrial anomalies linked to the development of atrial fibrillation (AF) in different cohort settings.

**Objective** To compare electrocardiographic parameters, such as P waves, in predicting long-term AF risk in acute ischemic stroke cases.

**Methods** The data of 231 consecutive acute ischemic stroke cases were retrospectively collected. Two independent cardiologists interpreted the electrocardiography recordings for PWPT, PWD, and P wave amplitude in lead DI. The median follow-up study period was 16 (interquartile range [IQR]: 11–24) months.

**Results** In total, AF was detected in 43 (18.6%) cases. All studied P wave parameters were found to be statistically significant in cases with AF. Based on multivariable logistic regression analysis, dementia, left atrium volume index, PWD (razão de chances [RC]: 1.11; 95% confidence interval [CI]: 1.058–1.184; *p* = 0.003), PWPT in lead DII (RC: 1.030; 95%CI: 1.010–1.050; *p* = 0.003), and advanced interatrial block morphology were independent predictors of long-term AF. P wave duration had the highest area under the curve value, sensitivity, and specificity for long-term AF in such cases compared with the other P wave parameters.

**Conclusions** Our head-to-head comparison of well-known P wave parameters demonstrated that PWD might be the most useful P wave parameter for long-term AF in acute ischemic stroke cases.

## Resumo

**Antecedentes** Parâmetros eletrocardiográficos, como tempo de pico da onda P (PWPT, na sigla em inglês), duração da onda P (PWD, na sigla em inglês) e amplitude da onda P na derivação DI, têm sido utilizados para avaliar anomalias atriais esquerdas ligadas ao desenvolvimento de fibrilação atrial (FA) em diferentes cenários de coortes.

**Objetivo** Comparar os parâmetros eletrocardiográficos destas ondas P na predição do risco de FA de longo prazo em casos de acidente vascular cerebral (AVC) isquêmico agudo.

**Métodos** Os dados de 231 casos consecutivos de AVC isquêmico agudo foram coletados retrospectivamente. Dois cardiologistas independentes interpretaram os registros eletrocardiográficos para PWPT, PWD e amplitude da onda P na derivação DI. O período médio do estudo de acompanhamento foi de 16 (intervalo interquartil [IQR, na sigla em inglês]: 11–24) meses.

**Resultados** No total, FA foi detectada em 43 (18,6%) casos. Todos os parâmetros da onda P estudados foram considerados estatisticamente significativos nos casos com FA. Com base na análise de regressão logística multivariável, demência, índice de volume do átrio esquerdo, PWD (razão de chances [RC]: 1,112; intervalo de confiança [IC] 95%: 1,058–1,184; *p* = 0,003), PWPT na derivação DII (RC: 1,030; IC95%: 1,010–1,050; *p* = 0,003) e avançada morfologia do bloqueio interatrial foram preditores independentes de FA de longo prazo. A PWD teve a maior área sob o valor da curva, sensibilidade e especificidade para FA de longo prazo em tais casos em comparação com os outros parâmetros da onda P.

**Conclusões** Nossa comparação direta de parâmetros da onda P bem conhecidos demonstrou que a PWD pode ser o parâmetro da onda P mais útil para FA de longa duração em casos de AVC isquêmico agudo.

## INTRODUCTION

Acute stroke, which is broadly classified into either of ischemic or hemorrhagic origin, is defined as new and rapid onset clinical findings of global or focal cerebral dysfunction that usually present for ≥ 24 hours. 1 Acute ischemic stroke (AIS), which accounts for ∼ 80% of all cases, is mainly caused by atherosclerosis of extracranial carotid arteries and, less commonly, by cardioembolism. 1 Remarkably, cardioembolic stroke is often more severe, extensive, and clinically significant when compared with stroke of atherosclerotic origin. 2 The most common cause of cardioembolic stroke is atrial fibrillation (AF), which is associated with a 5-fold increase in the risk for AIS. 2 3 Thus, identifying patients who present with AF is of utmost importance to appropriately manage the treatment regimens and to prevent the occurrence of future strokes. On the other hand, detecting AF is time-consuming and cumbersome for most patients, since it necessitates wearing an electrocardiography (ECG) Holter monitoring for an extended period or even an implantable loop recorder. 4 As a result, it becomes clinically apparent that this type of extended rhythm monitoring method should be selectively used to target the cases that are at the highest risk for AF.

Even in the absence of obvious AF, abnormalities in the left atrium (LA) are linked to an elevated risk of AIS and an ECG can be used to detect such abnormalities noninvasively. 5 Electrocardiographic parameters, such as P wave peak time (PWPT), P wave duration (PWD), and P wave amplitude in lead DI, have been utilized to assess left atrial anomalies linked to the development of AF in different cohort groups. 6 7 8 9 10 Nevertheless, there is a limited clinical data regarding the usefulness of these parameters in predicting the risk of AF in patients with AIS. Additionally, no prior study has tested the efficacy of these parameters to indicate which AIS patients are at the highest risk for the incidence of AF in the long-term.

The main purpose of the present investigation was to compare these P wave parameters in predicting long-term AF risk in AIS cases.

## METHODS

### Data gathering

Initially, patients who were diagnosed with AIS due to extracranial carotid artery stenosis were excluded from the study. Additionally, patients with moderate to severe heart valve disease and a history of cardiac valve surgery were excluded from the investigation. Then, data of consecutive AIS cases ( *n* = 272) who were admitted to our hospital between January 2017 and December 2020 were retrospectively collected. In total, 32 patients were excluded due to AF, 4 had a poor-quality ECG, 3 had an implantable device pacing the atrium, and 2 did not have an ECG ( *n* = 41). All cases were on sinus rhythm on admission and before hospital discharge. The diagnosis of AIS was confirmed by an experienced neuroradiologist using diffusion-weighted magnetic resonance imaging (MRI). Additionally, computed tomography angiography was applied to exclude significant carotid artery stenosis if a stenosis > 50% was detected on Doppler ultrasonography. Clinically relevant medical data was obtained from the hospital electronic health records and from the national health record data. The protocol and design of the investigation was approved by the Ethics Committee.

### ECG analysis and holter monitoring

**Figure 1:**
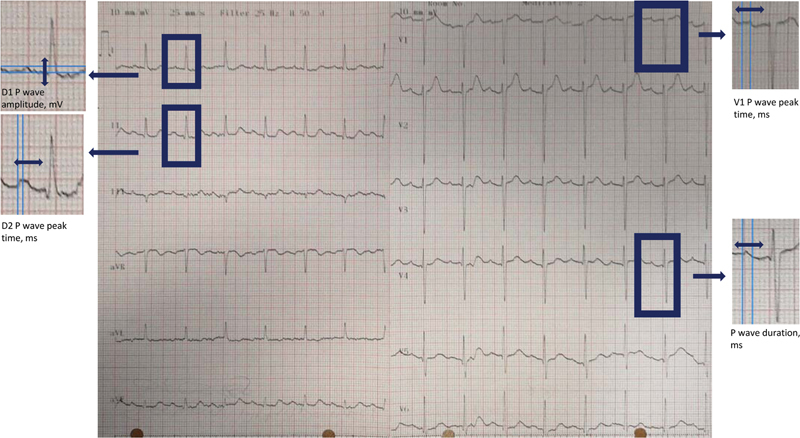
An example of electrocardiography showing how to measure the P wave duration (PWD), P wave peak time (PWPT) in lead DII and VI, and P wave amplitude in lead DI.

A conventional 12-lead ECG (Schiller Cardiovit AT-102-G2 machine) with 25 mV recording was obtained for each case included in the present study. All ECG recordings were digitalized with 300 dpi scanning and 10x amplification. Two independent cardiologists interpreted the ECG recordings. The EP Calipers (EP Studios, London, UK) application was used to measure the amplitudes and time periods in the ECG recordings. P wave peak time was accepted as the time from the isoelectric line to the peak of the P wave, and it was measured in lead DII and VI. P wave duration was accepted as the longest PWD across 12 leads, and it was measured as the time interval between the end of the T-P segment (the onset of the P wave) and the return to baseline (PR interval). In case of presence of biphasic P-wave, PWD was measured by including both positive and negative deflections from baseline. P wave amplitude was measured as the amplitude in lead DI. Intra- and interobserver coefficients of variation were 3 and 5% for PWPT, 3 and 4% for PWD, and 4 and 5% for P wave amplitude in lead DI, respectively. Prolonged PWD + biphasic P-wave shape in leads III and aVF with biphasic morphology or notched morphology in lead II were accepted as an advanced interatrial block. An exemplary ECG showing the measurements of PWD, PWPT in lead DII and VI, and P wave amplitude in lead DI is presented in Figure 1 .

Each patient was evaluated for AF with an ambulatory Holter rhythm monitoring (Schiller, Cardiovit AT-10 plus) for 72 hours. A 72-hour Holter rhythm monitoring was implemented in all AIS patients within 7 days of the index stroke event. Also, the data of the national health records were examined for each case for any AF diagnosis during the study period.

### Transthoracic echocardiography and laboratory examination

A cardiac imaging specialist performed echocardiographic examinations on each patient. The left ventricular (LV) ejection fraction was measured using the biplane Simpson method. LV and LA diameters were measured form the parasternal long-axis, apical 4-chamber, and apical 2-chamber views. The LA volume index was estimated using the biplane area-length method at the time of opening of the mitral valve and was corrected for body surface area.

Upon admission to our institution, all blood samples were collected from the antecubital vein. A Beckman Coulter LH 780 hematology analyzer and a Beckman Coulter LH 780 device (Beckman Coulter, Brea, CA, USA) were used to examine all blood parameters.

### Statistical analysis

Statistical analyses were performed using the IBM SPSS Statistics for Windows, version 20.0 (IBM Corp., Armonk, NY, USA). To determine the normality of the data, the Kolmogorov-Smirnov test was used. The median [interquartile range (IQR)] for continuous variables was used. Quantitative data was represented using absolute and relative frequencies. Continuous variables were compared using either the independent Student *t* -test or the Mann-Whitney U-test. We used either the Pearson chi-squared test or the Fisher exact test to assess the categorical data. Using univariable logistic regression analysis, the independent predictors of long-term AF were first identified. The parameters that were statistically significant in the univariable logistic regression analysis were then incorporated into a multivariable logistic regression analysis to determine the independent predictors of long-term AF. The cutoff value of each analyzed P wave parameter was determined using a receiver operating characteristic (ROC) curve analysis. The area under the curve (AUC) values of each P wave parameter were also compared by the DeLong method. A *p* -value < 0.05 was deemed significant in all statistical analyses.

## RESULTS

Finally, 231 AIS cases were analyzed in the present investigation, 150 (64.9%) of which were males. In total, AF was detected in 43 (18.6%) cases. The median follow-up study period was 16 (IQR: 11–24) months. The patients were categorized into two groups: those who developed or who did not develop AF in the long-term follow-up.

All baseline characteristics and echocardiographic findings of the cases are listed in Table 1 . Patients who had AF were older and the frequency of dementia was significantly greater among these patients. Other baseline characteristics were similar in both groups. For echocardiographic parameters, the two groups were different in terms of LA volume index and LA anteroposterior diameters.

**Table 1:** Baseline clinical characteristics and echocardiographic findings of all patients according to the development of atrial fibrillation in the long-term follow-up

		Atrial fibrillation in the long-term follow-up (-), ( *n* = 188)	Atrial fibrillation in the long-term follow-up (+), ( *n* = 43)	*P* value
Baseline characteristics	Age, years	66 (55–75)	74 (67–83)	0.003
	Gender, male, n (%)	121 (64.4)	29 (67.4)	0.701
	Hypertension, n (%)	138 (73.4)	32 (74.4)	0.891
	Diabetes mellitus, n (%)	69 (36.7)	20 (46.5)	0.308
	Insulin dependency, n (%)	14 (7.4)	6 (14.0)	0.224
	Hyperlipidemia, n (%)	82 (43.6)	15 (34.9)	0.292
	Smoker, n (%)	38 (20.2)	9 (20.9)	0.916
	Chronic renal failure, n (%)	19 (10.1)	8 (12.5)	0.598
	Coronary artery disease, n (%)	48 (25.5)	14 (21.9)	0.554
	COPD, n (%)	18 (9.6)	6 (14.0)	0.408
	Cancer, n (%)	10 (5.3)	3 (7.0)	0.713
	Dementia, n (%)	15 (8.0)	12 (27.9)	<0.001
Echocardiographic parameters	LV ejection fraction, %	60 (59–61)	60 (59–61)	0.654
	LV end-diastolic dimension, mm	46 (44–48)	45 (44–48)	0.568
	LV end-systolic dimension, mm	25 (24–32)	25 (24–32)	0.392
	LA anteroposterior diameter, mm	36 (35–39)	37 (35–43)	0.023
	LA volume index, mL/m ^2^	22 (21–26)	31 (24–34)	<0.001
	Follow-up, months	22 (14–24)	8 (6–13)	

Abbreviations: COPD, chronic obstructive pulmonary disease; LA, left atrium; LV, left ventricular.

Continuous variables are presented as median (interquartile range), nominal variables presented as frequency (%).

We also compared the laboratory and ECG findings of all patients, as indicated in Table 2 . Only creatinine levels were found to be considerably higher in AF patients. Other variables were comparable in both groups. When the ECG parameters were evaluated, all studied P wave parameters, including PWD, PWPT in lead DII and VI, and P wave amplitude in lead DI, were found to be statistically significant in cases with AF. The frequency of advanced interatrial block morphology was 25.0% ( *n* = 47) in patients without AF and 76.7% ( *n* = 33) in those with AF, respectively.

**Table 2:** Laboratory variables and electrocardiography findings of all patients according to the development of atrial fibrillation in the long-term follow-up

		Atrial fibrillation in the long-term follow-up (-), ( *n* = 188)	Atrial fibrillation in the long-term follow-up (+), ( *n* = 43)	*P* value
Laboratory variables	Hemoglobin, g/dL	12.9 (11.9–14.4)	12.7 (11.2–13.8)	0.149
	RDW, %	13.2 (12.7–14.3)	13.3 (12.5–14.7)	0.558
	WBC, cells/µL	8.4 (6.8–9.9)	8.8 (6.7–10.7)	0.391
	Platelet count, cells/µL	234 (184–286)	226 (194–288)	0.865
	MPV, fL	9.3 (8.2–10.1)	9.2 (8.2–9.8)	0.425
	Creatinine, mg/dL	0.9 (0.8–1.1)	1.1 (0.9–1.3)	0.018
	Urea, mg/dL	36 (26–47)	37 (31–54)	0.080
	TSH, nmol/L	1.2 (0.6–2.3)	1.0 (0.5–2.0)	0.446
	AST, U/L	20 (16–27)	20 (16–24)	0.526
	ALT, U/L	20 (14–32)	22 (13–25)	0.488
	Albumin, mg/dL	37 (32–41)	37 (35–39)	0.871
	Glucose, mg/dL	106 (88–146)	117 (86–168)	0.581
Electrocardiography parameters	P wave duration, ms	112 (98–121)	128 (120–142)	<0.001
	P wave peak time in lead DII, ms	55 (47–66)	64 (57–76)	<0.001
	P wave peak time in lead VI, ms	46 (37–55)	55 (45–64)	<0.001
	P wave amplitude in lead DI, ms	0.12 (0.10–0.14)	0.09 (0.08–0.13)	<0.001
	Advanced interatrial block, n (%)	47 (25.0)	33 (76.7)	<0.001

Abbreviations: ALT, alanine aminotransferase; AST, aspartate aminotransferase; MPV, mean platelet volume; RDW, red cell distribution width; TSH, thyroid stimulating hormone; WBC, white blood cell.

Continuous variables are presented as median (interquartile range), nominal variables presented as frequency (%).

Table 3 shows the findings of the univariable and multivariable logistic regressions. In univariable regression, age, dementia, LA volume index, creatinine, PWD, PWPT in lead DII and VI, P wave amplitude in lead DI, and interatrial block morphology were all linked to long-term AF. These parameters were then included into the multivariable logistic regression analysis. Dementia, LA volume index, PWD (OR: 1.112; 95% confidence interval [CI]: 1.058–1.184; *p* = 0.003), PWPT in lead DII (OR: 1.030; 95%CI: 1.010–1.050; *p* = 0.003), and advanced interatrial block morphology were all found to be independent predictors of long-term AF based on the multivariable logistic regression analysis.

**Table 3:** Univariable analysis and multivariable model for independent predictors of the long-term atrial fibrillation*

	Univariable analysis		Multivariable analysis	
	*P* value	HR (95% CI)	*P* value	HR (95% CI)
Age	0.009	1.032 (1.008–1.056)	−	−
Dementia	<0.001	3.762 (1.928–7.339)	<0.001	3.163 (1.689–5.923)
LAVI	<0.001	1.204 (1.159–1.250)	<0.001	1.120 (1.058–1.186)
Creatinine	0.024	1.625 (1.066–2.478)	−	−
P wave duration	<0.001	1.044 (1.028–1.060)	0.003	1.112 (1.058–1.184)
P wave peak time in lead DII	<0.001	1.058 (1.039–1.078)	0.003	1.030 (1.010–1.050)
P wave peak time in lead V1	<0.001	1.040 (1.026–1.054)	−	−
P wave amplitude in lead DI	0.002	0.862 (0.715–0.978)	−	−
Advanced interatrial block	<0.001	8.756 (4.302–17.822)	<0.001	9.274 (2.867–30.005)

Abbreviations: CI, confidence interval; HR, Hazard ratio; LAVI, left atrium volume index.

*All clinically relevant parameters were included in the model.

**Figure 2:**
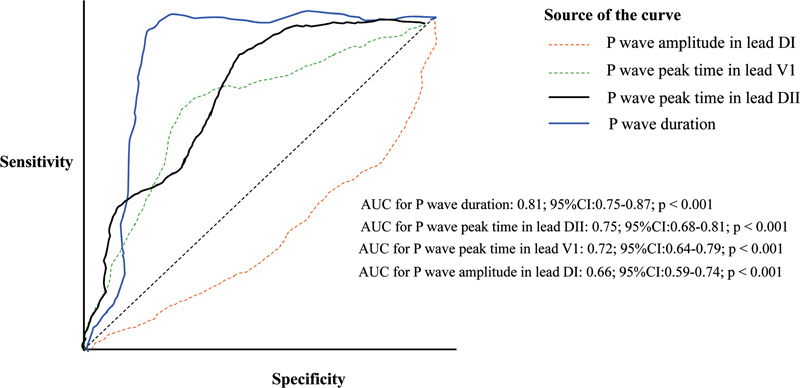
A receiver operating characteristic curve comparison of P wave duration (PWD), P wave peak time (PWPT) in lead DII and VI, and P wave amplitude in lead DI to predict long-term atrial fibrillation.

When the AUC value of each P wave parameter was compared with predicting long-term AF, we found that PWD had the highest value (AUC: 0.81; 95%CI: 0.75–0.87; *p* < 0.001), followed by PWPT in lead DII (AUC: 0.75; 95%CI: 0.68–0.81; *p* < 0.001), PWPT in lead VI (AUC: 0.72; 95%CI: 0.64–0.79; *p* < 0.001), and P wave amplitude in lead DI (AUC: 0.66; 95%CI: 0.59–0.74; *p* < 0.001) ( Figure 2 ). To predict long-term AF risk, the ideal value of PWD was > 122 milliseconds, with 76% sensitivity and 85% specificity; the ideal value of PWPT in lead DII was > 60 milliseconds, with 71% sensitivity and 62% specificity; the ideal value of PWPT in lead VI was > 50 milliseconds, with 68% sensitivity and 73% specificity; and the ideal value of P wave amplitude in lead DI was > 0.11 mV, with 68% sensitivity and 73% specificity.

## DISCUSSION

The present study was the first investigation to compare well-known electrocardiographic P wave parameters in predicting long-term AF in AIS cases. According to the results of the present study, we found that PWD had the highest AUC value, sensitivity, and specificity for long-term AF in AIS cases compared with the other electrocardiographic P wave parameters, including PWPT in lead DII, PWPT in lead VI, and P wave amplitude in lead DI. Additionally, only PWD and PWPT in lead DII were independently linked with long-term AF in AIS cases.

Currently, AIS is a significant cause of morbidity and mortality in developing and developed countries. 1 In patients who present with AIS, cardiac arrhythmias, especially AF, are one of the main underlying causes of stroke due to cardioembolism. 11 Thus, detecting AF and adopting an appropriate anticoagulation strategy can decrease dramatically the risk of stroke. 12 Specially, as a cheap and easy diagnostic test, the role of ECG in this task can decrease health-related costs and help focus on limited resources, like ambulatory Holter rhythm monitoring, where they are needed.

On ECG, P wave is indicative of atrial depolarization, which originates from the sinoatrial node in proximity to the right atrium (RA), follows the interatrial conduction pathways, and depolarizes the LA. 13 Any disturbances or modifications to this pathway may present themselves as a prolongation of PWD on the ECG, and studies have shown that prolonged PWD might represent the presence of atrial myopathy. In some studies, PWD had been linked to AIS. 5 8 Nevertheless, Kocer et al found that AIS patients and control subjects might have similar PWD. 14 Besides that, in a recent systematic review and meta-analysis of P wave indices, PWD was not found to be linked with AIS. 5 In our study, PWD was shown to be longer in AIS cases who had AF than in those who did not have AF. When compared with the other P wave parameters, PWD had the greatest AUC value for predicting AF over the long-term. Remarkably, PWD was also found to be independently associated with the presence of AF in patients with AIS. Furthermore, advanced interatrial block morphology was observed more frequently in the AIS patient group who developed AF.

Another ECG parameter investigated in the present study was PWPT. Although a normal P wave reflects depolarization of both atriums, the LA depolarization accounts for the majority of P wave amplitude since it has a larger mass than the RA. 13 As a result, the later the peak in the P wave can be noticed since it takes longer for the action potential to reach the LA. Yıldırım et al previously reported that even while PWPT in lead VI and DII were longer in the AF group, only PWPT in lead VI was a predictive of paroxysmal AF in patients with AF history. 9 By contrast, Burak et al found that only PWPT in lead DII was associated with more extensive coronary atherosclerosis in acute coronary syndrome patients. 10 However, there is a lack of data regarding PWPT for long-term AF risk in patients with AIS. In this study, we found that only PWPT in lead DII was an independent predictor of the long-term AF in AIS. On the other hand, its AUC value, sensitivity, and specificity for long-term AF were slightly lower compared with PWD.

Last electrocardiographic P wave parameter analyzed in this investigation was P wave amplitude in lead DI. As it was mentioned previously, most of the P wave amplitude results from left atrial depolarization. So, a higher P wave voltage in lead DI can mean that both atriums depolarize synchronously and normally. 15 Park et al previously showed that low P wave amplitude in lead DI was suggestive of misplaced interatrial conduction and could predict paroxysmal AF recurrence following catheter ablation. 6 Gorenek et al found that low P wave amplitude in lead DI can predict immediate recurrence of AF after external cardioversion. 16 Moreover, Alexander et al demonstrated that low P wave amplitude in lead DI was linked to new-onset AF in the ACS population. 17 In our investigation, this P wave index was not shown to be associated with long-term AF in AIS cases. In addition, when compared with PWD, PWPT in lead DII and VI, it had the lowest AUC value, sensitivity, and specificity for long-term AF.

In our study, although PWD and PWPT in lead DII has been found to be independent predictors of long-term AF, the clinical importance of them appears to lower compared with the LA volume index and interatrial block. Therefore, new clinical score systems can be formed to increase the clinical value of these ECG parameters.

## LIMITATIONS

Our investigation had some limitations. First, the study was retrospective in nature, which could be accepted as the major limitation. Second, because consecutive AIS patients from a single center were recruited, it might limit the applicability of the results to a wider population and create a predisposition to selection bias. But, this limitation might be alleviated by our relatively large study participants. Third, although a 72 hours Holter monitoring was applied for each case enrolled in the study, we did not utilize implantable loop recorders or wearable rhythm monitors, which could offer superior utility then the limited time analysis offered by ambulatory Holter rhythm monitoring. Finally, even though ECG recordings were anonymously analyzed by two expert cardiologists, computer analyses of the traces were not performed, and this might lead to human error in analyses.

In conclusion, our head to head comparison of well-known P wave indices demonstrated that PWD had the highest AUC value, sensitivity, and specificity for long-term AF when compared with the other electrocardiographic P wave indices, including PWPT in lead DII, PWPT in lead VI, and P wave amplitude in lead DI. In addition, we consider that AIS patients with prolonged PWD and PWPT in lead DII may be at the highest risk of AF during the long-term period. Thus, more vigilant follow up for AF among these patients should be commenced.
